# Trial feasibility and process evaluation of a motivationally-embellished group peer led walking intervention in retirement villages using the RE-AIM framework: the residents in action trial (RiAT)

**DOI:** 10.1080/21642850.2019.1629934

**Published:** 2019-06-17

**Authors:** Cecilie Thøgersen-Ntoumani, Eleanor Quested, Stuart J.H. Biddle, Marlene Kritz, Jenny Olson, Elissa Burton, Ester Cerin, Keith D. Hill, Joanne McVeigh, Nikos Ntoumanis

**Affiliations:** a Physical Activity and Well-Being Lab, School of Psychology, Curtin University, Perth, Australia; b Physically Active Lifestyles Research Group, University of Southern Queensland, Springfield, Australia; c School of Physiotherapy and Exercise Science, Curtin University, Perth, Australia; d Mary MacKillop Institute for Health Research, Australian Catholic University, Melbourne, Australia; e School of Public Health, The University of Hong Kong, Hong Kong, Hong Kong; f School of Occupational Therapy, Social Work and Speech Pathology, Curtin University, Perth, Australia; g Movement Physiology Laboratory, School of Physiology, University of Witwatersrand, Witwatersrand, South Africa

**Keywords:** Peer leaders, walking intervention, retirement villages, motivation training, older adults

## Abstract

**Objective**: The Residents in Action Trial (RiAT; ACTRN12616001177448) was a 16-week motivationally-embellished peer-led walking intervention designed to increase walking, reduce sitting, and improve mental health and well-being in insufficiently active residents in retirement villages. In this paper we report on 1) trial feasibility and acceptability, and 2) evaluate the processes involved in the implementation of the intervention using the RE-AIM framework.

**Method**: A mixed methods design was employed, consisting of data from accelerometers, surveys, (individual, pair-based and focus group) interviews, and participant logbooks. Participants included 116 walkers (*M*(*SD*) age = 78.37(8.30); 92% female), 8 peer leaders (i.e. ambassadors) and 3 retirement village managers from 14 retirement villages. Descriptives and linear mixed modelling were used to analyse the quantitative data and inductive thematic analyses were employed to analyse the interview data.

**Results**: The intended cluster randomised controlled design became quasi-experimental due to insufficient numbers of recruited ambassadors. The perceived burden of the number and frequency of research assessments was a frequently mentioned reason for a poor recruitment. Facilitators to walking maintenance were the use of self-monitoring, goal setting, social support, and having a routine. Reach was modest (about 14% of eligible participants were recruited from each village), but retention was excellent (92%). The motivational strategies taught appeared to have been implemented, at least in part, by the ambassadors. The walkers in the main experimental condition increased marginally their step counts, but there were no group differences on mental health and well-being outcomes, partly because of low statistical power.

**Conclusions**: Walkers and ambassadors who did take part in the study suggested that they enjoyed the programme and found it useful in terms of becoming more active and making social connections. However, the group format was not appealing to some participants, hence, other delivery options should be explored in the future.

## Introduction

Older adults represent a growing proportion of the population, and it is estimated that by 2050, 16% of the global population will be aged 65 and older (World Health Organisation, 2011). This development promises many societal benefits but also many challenges. Chronic disease and frailty are more likely to develop with age (Kennedy et al., 2014), and these outcomes are associated with increased risk for the poor quality of life (Kojima, Iliffe, Jivraj, & Walters, 2016), and loneliness (Barlow, Liu, & Wrosch, 2015). Physical activity can ameliorate these risks, but the majority of older adults do not meet national and international physical activity recommendations for health (Hallal et al., 2012).

A sizeable proportion of older adults relocate to retirement villages in many parts of the Western World, and given the changing demographics, the number of individuals moving into retirement villages is likely to increase. Retirement villages are housing developments which offer a range of accommodation options and diverse services and facilities for relatively healthy people aged 55 and over who have retired, or whose spouse has retired. In many retirement villages, exercise facilities and structured activities (e.g. swimming pools, gyms, and yoga classes) are available to residents in an effort to help them stay independent, mobile and well-functioning, both physically and psychologically. However, a large proportion of retirement village residents do not find these opportunities appealing (Thogersen-Ntoumani et al., 2017). In Western Australia, for example, it was found that only 27% of residents in a wide range of retirement villages met physical activity recommendations for health, as assessed via accelerometers (Nathan, Wood, & Giles-Corti, 2014).

### Walking to promote physical activity in older adults

For many insufficiently physically active older adults, walking is the preferred type of physical activity (Amireault, Baier, & Spencer, 2018). Walking performed in groups is particularly beneficial for a range of health indicators, including blood pressure, body mass index, fitness, depression and physical functioning (Hanson & Jones, 2015). Group-based walking is also particularly well suited for older adults as social interaction is a powerful motivator for this age group (Zubala et al., 2017).

### Theory-based interventions

Self-determination theory (SDT; Ryan & Deci, 2017) is a theoretical framework that may be particularly useful to understand determinants of sustained behaviour change. According to Ryan and Deci, satisfaction of three basic psychological needs, universal to all people, are key factors for optimal growth and well-being. These are the needs for autonomy (i.e. perceiving a sense of choice, ownership and control over one’s behaviour), competence (i.e. feeling capable of attaining desired outcomes), and relatedness (i.e. experiencing belonging and meaningful relations). When these needs are satisfied, individuals are more likely to experience high quality motivation, termed ‘self-determined’ motivation. In turn, self-determined motivation fuels continued sustained engagement in behaviours and leads to more desirable well-being outcomes. On the other hand, when these needs are not satisfied, people will develop low quality motivation, called ‘controlled motivation’ which leads to lower participation rates and ill-being. Much support for this theory exists, including in the physical activity and health domains (Ng et al., 2012; Teixeira, Carraca, Markland, Silva, & Ryan, 2012).

According to SDT, the social environment plays a pivotal role in determining the extent to which individuals will experience psychological need satisfaction, and therefore develop self-determined motivation, sustained engagement and optimal well-being. The communication style adopted by social agents, such as exercise instructors or healthcare professionals, can be described as need-supportive and/or need thwarting, depending on the predominant characteristics of the style adopted. A need-supportive style is characterised by features that support feeling of autonomy, competence and relatedness, including among other things, the provision of meaningful choice, competence-enhancing feedback and empathy or warmth. In contrast, someone who uses a predominantly need-thwarting style may offer little variety and limited or no choice, provide demeaning feedback or show no warmth or care towards the recipient. When a need-supportive style is adopted, the recipient is most likely to experience need satisfaction, while a need thwarting style will lead to the experience of need frustration (Ntoumanis, Edmunds, & Duda, 2009). Interventions have been developed in the physical activity domains to train social agents in positions of authority (e.g. healthcare professionals, teachers and exercise instructors), to adopt need-supportive interpersonal styles. Indeed, evidence has supported the feasibility of training such agents, and provided evidence that a need-supportive style will result in high quality motivation, sustained engagement and psychological well-being (e.g. Ntoumanis, Thøgersen-Ntoumani, Quested, & Hancox, 2017). However, there is a lack of evidence on the feasibility, and effects, of training individuals who are not traditionally in a position of authority, such as peers, to adopt a leadership role and provide need support to others.

### Peer-led approaches to intervention delivery

It has been argued that peer leaders may be particularly effective intervention agents, because they can facilitate attention, retention and motivation in recipients (Ginis, Nigg, & Smith, 2013). In part due to their similarities to target individuals, they may be seen as credible and empathic messengers in the promotion of physical activity. Indeed, a systematic review by Ginis et al. (2013) found that peer-based physical activity interventions were equally as effective as those delivered by professionals. Peer-based interventions are generally categorised as peer-delivered or peer-assisted (Matz-Costa, Howard, Castaneda-Sceppa, Diaz-Valdes Iriarte, & Lachman, 2018). A recent meta-analysis of 18 intervention studies (eight of which were peer-delivered and five using peer support) found that exercise programmes involving peers were effective in promoting programme engagement and maintaining adherence (Burton et al., 2018). To our knowledge, none of these peer-based interventions have trained peers to use SDT-based need-supportive strategies when delivering an intervention.

### Process evaluations of physical activity interventions

Process evaluations can enhance understanding of why specific interventions may or may not be effective and are particularly important in the evaluation of complex interventions with multiple interacting components. Process evaluations can help identify what works work for whom, how, and under what contextual conditions. For example, the UK Medical Research Council (MRC) guidance on process evaluation highlights issues of implementation (e.g. reach, fidelity), mechanisms of impact (e.g. hypothesised pathways to behaviour change), and context (e.g. contextual barriers and facilitators; Moore, Audrey, Barker, Bond, Bonell, Hardeman, & Baird, 2015).

Researchers have conducted process evaluations of physical activity trials using a range of approaches. One of the most common and widely used approach is the RE-AIM framework (Gaglio, Shoup, & Glasgow, 2013). The framework helps facilitate the development, delivery and evaluation of interventions through the five elements of Reach, Efficacy, Adoption, Implementation and Maintenance. The reach of the intervention refers to issues of recruitment and participation (e.g. numbers and representativeness); effectiveness refers to the outcomes of the intervention; adoption refers to the ‘number, proportion, and representativeness of settings and intervention agents (people who deliver the program) who are willing to initiate a program’ (see www.re-aim.org/about/what-is-re-aim/); implementation refers to whether the intervention was delivered as intended; maintenance refers to behaviour change maintenance and relapse (see Table 2 for definitions and more details).

### The residents in action trial (RiAT) intervention

The intervention evaluated in this paper was designed to increase walking, reduce sitting and improve mental health in physically inactive older adults residing in retirement villages in Western Australia (WA). The trial was proposed as a 16-week cluster randomised controlled design with residents from 14 retirement villages in WA. We aimed to recruit seven villages to be randomised to an experimental condition, in which physically active residents (i.e. peer leaders) would be trained to lead group walks for 10 of the 16 weeks, using need-supportive motivation strategies. Insufficiently physically active residents (new ‘walkers’) were the targeted participants for group walks in their villages. To support the peer leaders in identifying how to operationalise the motivational principles during the walks, their training introduced ten need-supportive ‘themes’ (see Table 1). These themes were not prescriptive but provided a potential foci or topics for each week of the programme which could help the peer leaders to implement need-supportive strategies known to develop high quality motivation, leading to sustained behaviour change. The walkers in this condition also attended a workshop, received a folder providing basic information about the current physical activity recommendations and the benefits of walking, a logbook, and motivation skills training. The remaining seven villages were to be randomly assigned to the control arm, in which peer leaders would also lead small group walks for 10 weeks but would not be motivationally trained. In this arm, new walkers received the same information about physical activity and the benefits of walking for health, but no motivation training. The specifics of the intervention protocol have been published previously (Thogersen-Ntoumani et al., 2017).

**Table 1.** Ambassador motivational strategies and evidence of their efficacy, implementation, and maintenance in the ambassadors plus motivation training condition.

Strategies peer leaders focused on in group walks	Efficacy	Implementation	Maintenance
Get to know the walkers and help them feel at ease (relatedness support)	‘*her husband who doesn’t get out much, he was great you know. I think I brought him out of himself a lot*’ (Val, 70, ambassador completer)	‘*I didn’t notice any motivational things, I just noticed that she [ambassador] walked with a few people just to socialise*’ (Beatrice, age 76, adherer)	
Help each walker feel like an important member of the group (relatedness support)	Social support ‘*I love walking but as I’m getting older, I’m finding that it’s getting a bit more and more difficult, but if you’ve got someone with you, it helps. I prefer to walk with someone if possible and I just thoroughly enjoy it the whole time*’ (Enid, age 86, adherer) ‘*I just need encouragement to keep going … I enjoyed the company. I felt good about it*’ (Henry, age 69, challenged but remained) *‘We were doing it in a team thing. I made sure that I’d be checking up on the others. ‘are you going to walk today? ‘are we going to walk today’? or if they are not, I’d say ‘Well I’m still going to walk’* (Sally, age 80, adherer)		
Help make walking more enjoyable (autonomous motivation)	Walking with others ‘*I mostly walk alone but I found it very entertaining to walk with other people. More socialising made the walk more enjoyable*’ (Elizabeth, age 87, adherer) ‘*I enjoyed the company. I felt good about it’* (Henry, age 69, challenged but remained)		
Help walkers feel successful (competence support)	*‘I think, [name of walker], when she first started, she sorts of lagged behind a bit because she is used to walking at a very slow pace … but she picked herself up … I walked with her the other day, and she said ‘Oh, I’m walking so much faster’. So, she felt very good about herself. You know she feels that she really achieved’* (Val, 70, ambassador, completer)		
Provide choice to walkers regarding their walking (autonomy support)		‘*We did have one little group that we got going but it was the group couldn’t walk very far with. We had to more or less [walk] partly indoors and then a few times we went around but still in the village, just walking around the streets here where they could sit if they needed to sit*’ (Anna, age 74, ambassador completer)	
Encourage input from walkers (autonomy support)		*‘ … when we knew that she [the ambassador] wasn’t gonna be around, we nominated somebody from the group, each day to be the ambassador and that was quite bit of fun’* (Rose, age 72, adherer) ‘*[name of ambassador] was my ambassador but then she went on holiday and then it all changed. Everybody just sort of took turns.’* (Anita, age 65, adherer) ‘*They had to go off cruising or whatever they were doing. There were 3 different ambassadors in our group*’ (Henry, age 69, challenged but remained)	
Explore what your walkers find useful in terms of reducing their sitting (autonomy support)	*‘The part where the interrupted sitting, I found quite interesting because a chiropractor had said to me before you said it that you shouldn’t sit any longer than half an hour. He actually said, when you’re sitting watching a TV programme at night, it’s very easy’* (Enid, age 86, adherer)		
Explore what your walkers find useful in terms of keeping up their walking (autonomy support and autonomous motivation)		Self-monitoring of behaviour – pedometer ‘*I still use it. As a matter of fact, I’ve got it on right now and it was a matter of great interest as to how many steps I’d done in one day and I try to increase them if I could’* (Elizabeth, age 87, adherer) Personal challenge promoted sustained motivation ‘*Keeping it ongoing was a challenge in itself because that’s what I do. I said, ‘Okay, I’m going to do it, so I’ve got to do it,’ and no matter what else comes up, you try*’ (Anita, age 65, adherer)	Self-determined motivation ‘*There was a group that did enjoy walking, that still keep going*’ (Anna, age 74, ambassador completer)
Explore what will help your walkers to continue their walking pattern after week 10 (autonomous motivation)			Self-monitoring (general) ‘*The other thing you might be interested is that I’m now using the apps on my phone too so that I keep counting steps*’ (Henry, age 69, challenged but remained) Social support ‘*I felt on the whole our group was very successful. We seem to have no trouble of meeting and we didn’t usually tell people why we wouldn’t be there, but I think we’re going to keep the group going*’ (Enid, age 86, adherer) ‘*Try and keep it going but open it up to other people in the village*’ (Beatrice, age 76, adherer) Personal drive to continue ‘*Even though I’ve finished the 16 weeks I’m just going to keep going for myself, walking bigger dogs*’ (Anita, age 65, adherer)
Celebrate success, discuss which changes your walkers have noticed as a result of the programme, and setting new goals (competence support)			Walking programme as a catalyst for new PA behaviours ‘*I must admit that since we did that group walk, the group and quite a few of those people do the circuit training group twice a week*’ (Anna, age 74, ambassador completer)

### Study aims

The aims of the present study were to (1) examine trial feasibility and acceptability of the RiAT intervention, and (2) evaluate the processes involved in the implementation of the RiAT intervention using the RE-AIM framework.

## Methods

### Design

**Figure 1.**
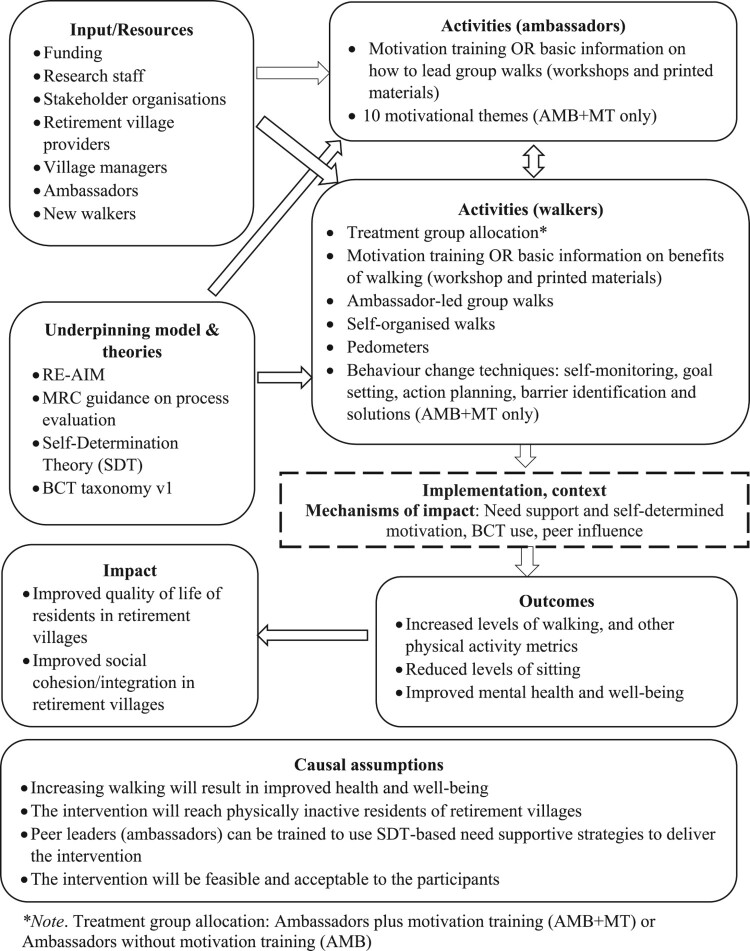
Logic model. *Note. Treatment group allocation: Ambassadors plus motivation training (AMB + MT) or Ambassadors without motivation training (AMB).

A mixed-methods design was employed, with data collected from accelerometers, surveys, individual, pair-based and focus group interviews, and participant log books. The definitions, data sources and operationalisation of each of the dimensions of the framework (Reach, Efficacy, Adoption, Implementation and Maintenance), as they relate to the present study, are provided in Table 2. Trial feasibility and acceptability were examined separately to the RE-AIM dimensions, because the former related to the trial procedures rather than the intervention per se. A logic model outlining input/resources, activities, mechanisms of impact, outcomes and impact are presented in Figure 1. Approval was sought and obtained from the host institution’s ethical review board, and the trial is registered with the Australian New Zealand Clinical Trials Registry (ACTRN12616001177448).

**Table 2.** Definitions, data sources and operationalisation of RE-AIM dimensions.

	Definition	Data sources	Operationalisation
Reach	Proportion and representativeness of eligible participants from partaking in the intervention	Data from age care providers, retirement village managers, interviews	N of walkers consenting to participate relative to those expressing initial interest N of consenting participants in relation to total N of residents per village who live in independent living units (information provided by village managers) N of walkers insufficiently physically active in relation to estimated eligible proportion Recruitment strategies used
Efficacy	The extent to which the intervention achieved its goals	activPAL monitor, questionnaire surveys, interviews	Attrition and drop-out (walkers) N and nature of adverse events Pre to post-intervention changes in PA and sitting (activPALs) Pre to post-intervention changes in general health and functioning, quality of life, anxiety and depression, loneliness and subjective vitality Qualitative data on effectiveness (post-intervention interviews with walkers and ambassadors)
Adoption	Proportion of aged care providers, villages and ambassadors taking part	Logs completed by researchers, questionnaire surveys, interviews	N of age care providers that agreed to participate in the trial N of villages per aged care provider agreeing to participate N of ambassadors expressing interest N of ambassadors consenting to participate N of ambassadors retained vs dropped out N of ambassadors completing training (face to face vs DVD) Satisfaction with ambassador training Extent to which ambassadors liked, felt confident and felt effective
Implementation	The extent to which the intervention was implemented as intended	Logs completed by researchers and walkers, surveys, interviews	N of walkers completing training (face to face vs DVD) Walkers’ ratings of acceptability of training Walkers’ ratings of acceptability of the whole intervention N of intervention tools used by walkers
Maintenance	The extent to which aspects of the intervention is maintained following the end of the intervention	Interviews	Extent to which group walks continued post-intervention Qualitative data on whether participants have kept up walking post intervention, from managers, ambassadors and walkers

### Participants

Inclusion criteria to participate as a ‘new walker’ included being a permanent resident of a retirement village in Western Australia, aged 60 and above, being able to communicate well in English, ability to provide consent and participate in baseline assessments, having no terminal illness or health problems that prevented walking (we included those who used walking sticks and walking frames), no known dementia diagnosis, could walk continuously on a flat surface at a light/moderate pace for at least fifteen minutes, had experienced fewer than two falls in the past 3 months, and self-reported that they did *not* currently meet the specific Australian Government guideline for older adults to accumulate at least 30 min of moderate intensity physical activity on most days of the week (Sims, Hill, Hunt, & Haralambous, 2010). The eligibility age to reside in retirement villages in Western Australia is predominantly 55 years and over. In order to avoid too many residents being ineligible for the study, the age criterion was set to 60 years and over as the main objective was to increase physical activity for people residing in retirement villages. Peer leaders were referred to as ‘ambassadors’ and were residents of retirement villages who met the PA guidelines and had an interest in becoming an ambassador. Other inclusion criteria for ambassadors matched those for the walkers.

### Data collection and assessment procedures

#### Trial feasibility and acceptability

To assess trial feasibility and acceptability, we recorded the number of villages that could be successfully randomised to their assigned treatment conditions. In addition, when interviewing village managers, ambassadors, and walkers we included questions to assess acceptability of the trial procedures and assessments.

##### Individual interviews

Semi-structured individual telephone interviews with walkers (*n *= 10) and ambassadors (*n *= 3) were conducted at the end of the intervention. We used purposive sampling with the walkers to include some participants who did not experience any personal or programme-related challenges during the intervention (Category A: adherers; *n *= 6), some who experienced either personal or programme-related challenges but who managed to overcome these challenges (Category B; challenged but remained; *n *= 3), and one other person who experienced personal challenges (health problems) that led them to drop out of the intervention (Category C; drop-out: *n *= 1). The interviews took between 30 and 45 minutes. The interview questions focused on reasons for participating in the intervention, challenges experienced during the trial, personal accounts of the effects of the intervention on behaviour, health and well-being, and current beliefs about the intervention.

Semi-structured phone interviews with managers from three different retirement villages explored perspectives on recruitment strategies, perceptions of engagement of the residents and the impact of the intervention on the residents who took part, factors influencing sustainability of the intervention beyond the research period, and whether residents were observed to continuing their walking, post-intervention.

##### Pair-based and focus group interviews

One pair-based and one structured focus group interview (*n *= 5) were conducted with other participants in two different villages. The pair-based interview consisted of two participants who had dropped out of the intervention. This interview was planned as a focus group interview but became pair-based because fewer than the anticipated number of participants attended the interview. The focus group interview was conducted with three adherers and two walkers who had been challenged but remained in the intervention. The participants were asked to reflect on reasons for signing up to the intervention, and their perceptions about the various aspects of the intervention, including the pedometers, the workshops, and the intervention materials. The participants were also asked if they had suggestions for improving the intervention.

To avoid duplication, below we elaborate on information not provided in Table 2.

#### Reach

To assess the extent to which we were successful in recruiting a representative proportion of insufficiently physically active residents (i.e. walkers), we estimated the number of participants who were indeed physically inactive at baseline (assessed via an activPAL micro3 device). Additionally, the percentage of walkers insufficiently physically active in relation to the estimated eligible proportion was estimated using existing data from retirement village residents in WA. This showed that 27.1% of residents were sufficiently physically active as measured via accelerometry (Nathan et al., 2014). As a result, we estimated that 72.9% of total residents across villages would be insufficiently physically active, and we compared the numbers we recruited in relation to this estimate. In accordance with Tudor-Locke et al. (2011), we operationalised insufficiently physically active participants as those individuals who took less than 7,500 steps on average per day at baseline; this was assessed from the step count data from the ActivPAL device.

#### Efficacy

##### Physical activity

Participants were provided with an activPAL micro3 device to wear for 7 days during baseline, 16 week follow up and 6 months. Participants were fitted with the device (by the research assistant), placed against the skin on the front of the right thigh halfway between the kneecap and pelvis. The device was waterproofed by wrapping it in a nitrile sleeve, allowing for 24-hour measurement. The devices had been initialised prior to begin recording at midnight following the measurement session. The time-stamped ‘event’ data file from the activPAL software (version 7.2.38) was used to determine time spent sitting/lying, standing, and stepping per day. The activPAL default MET values for sitting/lying and stepping events were used and METs for stepping events were estimated using the internal activPAL algorithm, which is based on a cadence-based linear regression. The event data file was then exported as a .csv file for further cleaning and analysis in R using a customised R programme (Process.AP; Lyden, Keadle, Staudenmayer, & Freedson, 2017) to estimate sedentary behaviour and other physical activity metrics (i.e. step counts, stepping time, light physical activity (LIPA), moderate-vigorous physical activity (MVPA), standing, and sitting time). Total MVPA (min/d) was computed as the sum of time spent in MVPA (stepping events > 3.00 METs) and LIPA (min/d) was computed from standing and stepping events with a MET value between 1.50–2.99 METs.

##### Mental health and well-being

###### General health and functioning

General physical and mental health over the last 4 weeks was assessed on the widely used SF-12 (Gandek et al., 1998). An example item was ‘how much did pain interfere with your normal activities?’. A physical health composite score (PCS) and a mental health composite score (MCS) were computed, with higher scores indicating better functioning. High levels of reliability and validity of the SF-12 have been reported in research with older adult populations (Gandek et al., 1998).

###### Quality of life (Dartmouth CO-OP charts)

Quality of life was assessed on the Dartmouth CO-OP charts (Nelson et al., 1987). This scale includes nine quality of life domains (i.e. physical fitness, feelings, daily activities, social activities, pain, change in health, overall health, social support, and quality of life). Participants are asked to respond reflecting on the past four weeks. For example, ‘During the past 4 weeks, how much have you been bothered by emotional problems such as feeling anxious, depressed, irritable or downhearted and sad?’. Items are rated on a scale ranging from 1 to 5, with higher scores indicating a greater quality of life. A previous study with a sample including older adults reported test-retest correlation coefficients between 0.93 and 0.99 (Lane, Carroll, Ring, Beevers, & Lip, 2001).

###### Anxiety and depression

Anxiety and depressive symptoms were assessed using the Hospital Anxiety and Depression Scale (HADS; Zigmond & Snaith, 1983). The scale comprises 14 items, seven of which assess anxiety (e.g. ‘I can sit at ease and feel relaxed’) and seven which measure depression (e.g. ‘I still enjoy the things I used to enjoy’). The items are scored on a 4-point scale ranging from 0 to 3, with higher scores indicating higher anxiety and depression. The psychometric properties of the HADS have been widely supported in previous research (Bjelland, Dahl, Haug, & Neckelmann, 2002).

###### Loneliness

Perceptions of loneliness were measured using three items, which have been psychometrically validated by Hughes, Waite, Hawkley, and Cacioppo (2004). An example item is ‘how often do you feel you lack companionship’ with responses ranging from *hardly ever,* to *often*. High scores indicate high levels of loneliness.

###### Subjective vitality

Feelings of energy were assessed using the Subjective Vitality scale (Bostic, McGartland Rubio, & Hood, 2000), which comprises of six items (e.g. ‘I feel alive and vital’). Responses range from 1 (*not at all true*) to 7 (*very true*). High scores indicate high levels of energy available to the self. Support for the scale’s psychometric properties has been reported by Bostic et al. (2000).

#### Adoption

Satisfaction with training was assessed at the end of the training using an adapted 9-item questionnaire developed by Hancox, Thøgersen-Ntoumani, Quested, and Ntoumanis (2018). The questionnaire was developed using Bowen and colleagues’ (2009) acceptability framework. Specifically, the survey assessed satisfaction with the training (e.g. ‘I enjoyed the workshops’), intention and motivation to use the strategies taught in the workshops (e.g. ‘The strategies I learnt in the workshops will be beneficial for the walkers in the RiAT program’), confidence (e.g. ‘I feel confident to use the strategies I have been taught in the workshops’), and perceived appropriateness (e.g. ‘I would recommend this workshop to other people’). Responses were rated on a scale ranging from 1 (*strongly disagree*) to 7 (*strongly agree*). Higher scores on the scale reflect high levels of training acceptability. The scale was internally reliable (*α* = .93). At the end of the intervention period, ambassadors also responded to items regarding whether they liked being an ambassador (responses ranging from *1 = dislike a great deal* to *5 = like a great deal*), and how confident (responses ranging from *1 = not confident at all* to *5 = extremely confident*), and effective (responses ranging from *1 = not effective* to *5 = extremely effective*) they felt as ambassadors.

#### Implementation

Information was retrieved from the walker log-books as to how many participants (from the relevant groups) used the intervention tools and use of these tools as a percentage of recommended frequency of use (i.e. self-monitoring of step counts, action planning sheets).

#### Training and intervention acceptability

The motivational training of the walkers was evaluated using a version of the 9-item questionnaire described earlier for evaluation of the training workshops for ambassadors, with the wording of some items amended slightly. The scale was internally reliable (*α* = .82). To assess acceptability associated with the intervention as a whole, we administered the same 9-item scale with the items referring to the ‘program’ rather than the ‘workshop’. An internal reliability coefficient of *α* = .86 was observed for this version of the scale.

### Data analyses

#### Quantitative data

Descriptive statistics (frequencies, *M*, *SE*) were calculated. We conducted linear mixed modelling with three levels: level 1 was time, level 2 represented individuals and level 3 was the villages. We assessed changes in outcomes over time by treatment condition (baseline to post-intervention or to 6-month follow-up depending on the availability of the data). The main analyses were conducted on an intention-to-treat basis. All analyses were conducted using SPSS 25.

#### Qualitative data

Thematic analysis (Braun, Clarke, & Weate, 2016) was used to analyse the data from all the interviews. This involved a series of six analytical phases. During phase 1, we familiarised ourselves with the data through repeated readings of the interview transcripts. In phase 2, we coded the data in the transcripts for content on a line-by- line basis; we used semantic coding, which captures descriptively the original (raw) data. In phase 3, the semantic codes were grouped, then subsequently developed as preliminary latent themes. Specifically, we grouped the semantically coded chunks with other similar ones, to develop preliminary themes that would map onto the RE-AIM framework. For example, we grouped together quotes that illustrated the degree to which the ambassadors implemented the motivational strategies under ‘implementation’. Phase 4 involved us scrutinising the themes identified in phase 3, in light of the original transcripts to ensure the themes were authentic and rooted in the data. Themes and sub-themes were subsequently generated in phase 5, with phase 6 involving the documentation of the themes. During phase 5, we drew from SDT and sought to identify examples of experiences of need support and experiences of different types of motivation (see Table 1). Specifically, we were interested to explore whether there was any evidence of use and impact of the 10 SDT-based, weekly need-supportive motivational strategies that the ambassadors had been taught. We scrutinised the themes and corresponding quotes and mapped relevant ones onto the motivational strategies. The motivational strategies are presented in the left column in Table 1. Two members of the research team independently coded the data and grouped the codes into possible themes. The coders discussed the themes, and any discrepant interpretations were resolved by consensus.

Qualitative data was used to explain quantitative findings, and to help enhance understanding of the contextual factors that shaped individual experiences. The integration of the quantitative and qualitative results was done using Fetters, Curry, and Creswell’s (2013) integrating through narrative ‘weaving approach’ whereby quantitative and qualitative findings were reported on a theme-by-theme basis.

## Results

Demographic characteristics of the walkers taking part in RiAT are presented in Table 3. Further, Table 4 provides an overview of the internal reliability coefficients and descriptives for the variables at each time point. All names reported are pseudonyms.

**Table 3.** Demographic characteristics of walkers in RiAT.

Variable	
Age years; mean (SD)	78.37 (8.30)
Female, n (%)	92 (79.30)
Years living in retirement village; mean (SD)	6.67 (5.15)
Marital status; married, n (%)	51 (44)
Marital status; never married, n (%)	9 (7.8)
Marital status; widowed, n (%)	35 (30.20)
Marital status; separated or divorced, n (%)	16 (13.80)
Marital status; other, n (%)	1 (0.9%)
Number of chronic health conditions; mean (SD)	.97 (.94)
Type of tenancy; rental/subsidised, n (%)	12 (10.30)
Type of tenancy; subsidised, n (%)	11 (9.50)
Type of tenancy; lease for life, n (%)	66 (56.90)
Type of tenancy; resident funded and lease for life, n (%)	25 (21.60)
Happiness living in village (1 = very unhappy; 5 = very happy); mean (SD)	3.95 (1.34)

**Table 4.** Internal reliability coefficients and descriptives for walkers for each assessment timepoint.

Variable	Baseline		Post		6-month follow-up	
	*α*	*M(SD)*	*α*	*M(SD)*	*α*	*M(SD)*
Waking wear time (hrs/day)	–	15.07 (1.51)	–	14.74 (1.41)	–	14.18 (1.23)
Step counts	–	6170 (2461)	–	6920 (2255)	–	6456 (1451)
Stepping time (mins/day)	–	80 (29)	–	88 (24)	–	91 (21)
LIPA (mins/day)	–	273 (100)	–	294 (65)	–	266 (62)
MVPA (mins/day)	–	48 (20)	–	56 (19)	–	54 (12)
Standing (mins/day)	–	244 (92)	–	264 (58)	–	248 (55)
Sitting (mins/day)	–	566 (119)	–	534 (88)	–	565 (127)
PCS	–	42.22 (9.93)	.	42.11 (12.21)	–	40.87 (12.07)
MCS	–	51.35 (8.42)	–	51.51 (9.69)	–	48.15 (9.70)
Physical fitness	–	3.10 (1.14)	–	2.96 (.91)	–	2.68 (.96)
Emotional functioning	–	4.05 (.81)	–	4.16 (.92)	–	4.09 (.96)
Daily activities	–	4.28 (.81)	–	4.18 (.83)	–	4.34 (.75)
Social role functioning	–	4.40 (.69)	–	4.51 (.76)	–	4.61 (.87)
Perceived pain	–	3.81 (.99)	–	3.90 (1.03)	–	3.52 (1.15)
Change in health	–	3.68 (.89)	–	3.55 (.76)	–	3.27 (.59)
Overall health	–	3.79 (.82)	–	3.68 (.83)	–	3.57 (.95)
Social support	–	4.04 (1.04)	–	4.05 (1.02)	–	4.21 (1.12)
Quality of life	–	4.08 (.67)	–	4.04 (.65)	–	4.12 (.59)
Anxiety	.82	1.85 (.55)	.81	1.90 (.55)	.81	1.57 (.45)
Depression	.77	1.66 (.50)	.77	1.66 (.50)	.44	1.44 (.27)
Loneliness	.89	2.36 (.52)	.84	2.40 (.50)	.88	2.29 (.50)
Subjective vitality	.81	4.61 (1.18)	.81	4.42 (1.00)	.89	4.83 (1.17)

### Trial feasibility

**Figure 2.**
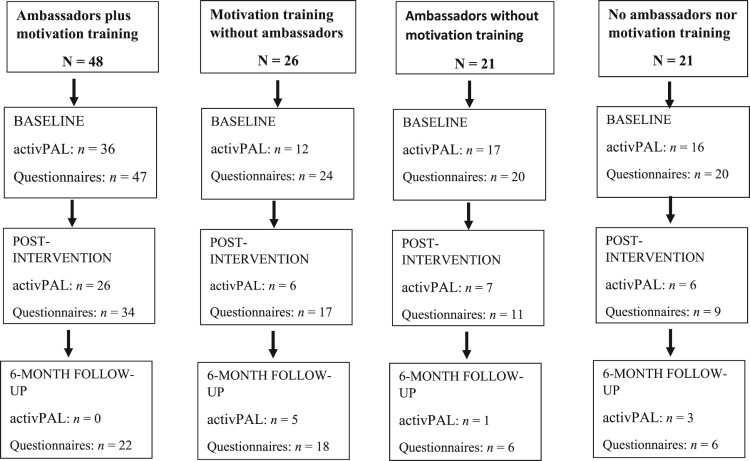
Flow of participant responses per condition.

Of the seven retirement villages originally assigned to the experimental condition (where both ambassadors and walkers were motivationally trained), three successfully completed this condition. In the remaining four villages, we did not manage to recruit residents who were willing to commit to the ambassador role. To achieve a balanced design across the 14 villages which had already been recruited, and taking into account the number of participants recruited across each village, we modified the design to consist of four different treatment conditions: (1) our original intervention arm, i.e. motivationally trained ambassadors and motivationally trained walkers (‘ambassadors plus motivation training’ AMB + MT; *n *= 3 villages), (2) motivationally trained walkers without ambassadors (‘motivation training without ambassadors’; MT; *n *= 3 villages), (3) our original control arm, i.e. ambassadors and walkers without motivation (‘ambassadors without motivation training’; AMB; *n *= 3 villages), and (4) no ambassadors with walkers who were not motivationally trained (‘no ambassadors nor motivation training’; No AMB or MT; *n *= 5 villages). As a result, the research design became quasi-experimental. The flow of participant responses to all assessments at each stage of the intervention by condition is illustrated in Figure 2. Due to the low numbers for the physical activity (activPAL) data at post-intervention in three out of four conditions, we only analysed the data from the participants in the AMB + MT condition from baseline to post-intervention. Additionally, we were unable to include any of the 6-month follow-up data for any condition for the same reasons.

Completing questionnaires as part of the research protocol was identified as a barrier to trial participation for some ambassadors. In the interviews, two of the managers and two of the ambassadors commented on the challenges associated with completing this ‘paperwork’. Bernadette stated:

The program was very well run but I just thought that there was far too much paperwork … (Bernadette, age 81, dropped out due to injury)

Some walkers also reported that there were too many questionnaires to complete. We also became aware that some residents joined the walking groups without wanting to participate in the trial because they did not want to complete any ‘paperwork’. Conversely, however, other walkers expressed their willingness to participate in recognition of the great value of the research:

I was most interested that you’d chosen to do it and chosen to work with elderly people. I thought that was great. I was pleased to be part of it. (Isobel, age 84, challenged but remained, AMB + MT)

Further, one participant noted that:

You coming down from [name of university] made us feel valued and useful to your project. (Leo, age 69, challenged but remained, AMB + MT)

However, some participants in the AMB + MT group wanted more contact with the research team, which might have impacted their continued engagement:

I would’ve liked to have had more contact with you girls. (Enid, age 86, adherer, AMB + MT)

Some participants expressed a misunderstanding of what was involved with participation in the study. For example, one lady who dropped out of the programme noted:

There was not enough communication about the actual study. It was just a walk study, and we said ‘yeah that would be great’ but then we came, and we were given those folders. (Harriet, female, age 68, drop-out, no AMB or MT)

Finally, views on the length of the 16-week intervention were mixed with some participants noting that they were ‘fed up’ by the end and that it was too long, while others indicated that they did not mind the length. Thus, it was clear through this qualitative data that for some participants, perceived challenges associated with participation in the research element of the study negatively impacted recruitment and (continued) involvement in the study.

### Reach

Interest in participating in the trial was expressed by 156 potential new walkers. Of those, 116 signed the consent form (74%) and 111 (71%) provided baseline data. The proportion of consenting participants across the 14 recruited villages ranged from 1-32% (average = 14%) of the total number of residents in independent living units in those villages. The proportion of insufficiently active participants (as assessed at baseline via the ActivPAL) was 70%. This rate is very similar to the rate (73%) reported by Nathan et al. (2014).

Recruitment strategies included coffee mornings with an information session about the study, promotional flyers, posters on village noticeboards, word of mouth, an information stand at a health fair, a study description published in village newsletters, letterbox drops, advert in a property council newsletter, and face to face meetings with village managers. It proved impossible to estimate how many participants had been recruited using each of these mechanisms. However, recruitment of the village with the largest number of participants was achieved through a resident ‘advocate’ (who sat on the resident committee in the village) who had seen the study advertised in the property council newsletter and contacted the research team.

One manager commented on the general lack of engagement of many of their residents, not only with regard to the present intervention, but in most activities happening in the villages:

I’ve got some residents who are very private residents that go off and do their own thing, don’t get involved in a lot, but then I have another, say 20 percent of the residents who would want to be involved. (Charlotte, manager, MT)

She also noted that some of the younger residents did not want to mix with older residents in the village by doing activities together. One of the walkers who was challenged but remained in the intervention suggested:

People come here to retire and sometimes that means just giving up and just doing what they want to do and nothing else. (Mary, age 81, challenged but remained, AMB)

These attitudes may have impacted the extent to which a large proportion of the residents in each village were engaged via the recruitment strategies we used. Further, ambassadors and walkers also perceived that many of the residents who were in the greatest need of the programme were not reached. For example, as expressed by one ambassador:

Unfortunately, the people [who you] are always hoping would join in and walk with us, they never did so, and they’re the people who really I thought would be the ones that should’ve been spoken to more independently, get them involved, because all of them just sit around and do nothing, and they’re able to walk but they need the motivation. (Bernadette, age 81, ambassador, drop-out, AMB + MT)

In contrast, Bev, an ambassador in another village, believed the programme had benefitted those in need, when she described a married couple taking part:

It was really great that the people who joined, especially a couple of them who probably wouldn’t have walked or socialised very much … I found that [the program was] advantageous to them … [Her] husband doesn’t get out much and do stuff, he was great … and he has continued to do it which is great. (Bev, age 70, ambassador, AMB + MT)

The importance of having programme advocates in the villages to get others interested was highlighted by several walkers:

I think probably if you were to do it again, maybe recruit a couple that – like for a start, if I knew, I would probably encourage a few more people to come along. (Selma, age 73, adherer, no AMB or MT)

### Efficacy

#### Attrition

Of the 116 new walkers who signed the consent form, 9 (8%) dropped out, five (four from AMB + MT and one from AMB) at the pre-intervention stage, and four (two from MT, one from AMB, and one from No AMB or MT) mid-intervention. Reasons included health problems (*n *= 4), perceived lack of time (*n *= 2), caring responsibilities (*n *= 1), personal circumstances (*n *= 1) and feeling that they could not comply with the requirements of the study (*n *= 1).

#### Adverse events

No adverse events were reported as a result of participation in the intervention.

#### Baseline differences between groups

We did not test for baseline differences between groups as this practice, despite its widespread use, is inappropriate (e.g. Altman & Doré, 1990; de Boer, Waterlander, Kuijper, Steenhuis, & Twisk, 2015).

#### Changes in outcomes

##### Physical activity and sitting behaviour

The results of the linear mixed modelling analysis for physical activity and sitting outcomes in the participants in the main experimental group (AMB + MT) are presented in Table 5. The baseline levels of MVPA were relatively high. The participants in this condition marginally increased their daily steps and they significantly increased the time they spent in stepping activities on a daily basis as a result of the intervention.

**Table 5.** Results of linear mixed modelling for physical activity and sitting behaviour in the ambassador plus motivation training condition, adjusting for village clustering.

Variable	Baseline *M (SE)*	Post *M (SE)*	*p*
Steps (per day)	7088 (649)	8077 (461)	.05
Stepping time (mins/day)	94 (8)	106 (5)	.04
LIPA (mins/day)	317 (28)	329 (14)	.41
MVPA (mins/day)	56 (6)	65 (4)	.07
Sitting (mins/day)	540 (29)	537 (23)	.89
Standing (mins/day)	280 (25)	292 (13)	.36

Note. LIPA = Light intensity physical activity; MVPA = Moderate to vigorous intensity physical activity.

##### Mental health and well-being

The results pertaining to changes in all mental health and well-being outcome variables across all conditions are presented in Table 6. Apart from a marginal reduction in the physical health composite score of the SF-12 (PCS) in the MT condition from baseline to post-intervention, there were no significant effects. Given the number of multiple comparisons, any p values below 0.05 should be treated with caution as they are likely to reflect Type I error.

**Table 6.** Results of linear mixed modelling mental health and well-being outcomes from pre to post-intervention across all conditions, adjusting for village clustering (using effect coding).

Variable	Baseline *M (SE)*	Post *M (SE)*	*p*
*PCS*			
Whole sample	42.37 (1.32)	42.21 (.72)	.83
AMB + MT	40.14 (3.61)	38.49 (1.98)	.41
MT	39.95 (4.18)	35.42 (2.29)	.05
AMB	35.43 (4.24)	34.04	.55
*MCS*			
Whole sample	51.64 (1.09)	51.81 (.80)	.84
AMB + MT	50.16 (2.99)	50.36 (2.20)	.62
MT	54.01 (3.47)	54.53 (2.55)	.84
AMB	50.08 (3.52)	49.85 (2.58)	.93
*Physical fitness*			
Whole sample	3.00 (.10)	2.92 (.10)	.46
AMB + MT	3.57 (.28)	3.28 (.27)	.28
MT	3.17 (.31)	3.31 (.31)	.65
AMB	2.43 (.32)	2.67 (.32)	.45
*Emotional functioning*			
Whole sample	4.03 (.08)	4.11 (.08)	.35
AMB + MT	4.14 (.22)	4.21 (.21)	.75
MT	4.32 (.25)	4.41 (.24)	.71
AMB	3.74 (.26)	4.07 (.25)	.19
*Daily activities*			
Whole sample	4.29 (.08)	4.17 (.08)	.15
AMB + MT	3.91 (.22)	3.99 (.22)	.72
MT	3.92 (.25)	3.94 (.25)	.94
AMB	3.72 (.26)	3.71 (.26)	.98
*Social role functioning*			
Whole sample	4.41 (.07)	4.50 (.08)	.29
AMB + MT	4.12 (.19)	4.21 (.21)	.67
MT	3.99 (.22)	4.34 (.24)	.15
AMB	4.08 (.22)	4.37 (.25)	.25
*Perceived pain*			
Whole sample	3.79 (.10)	3.90 (.09)	.25
AMB + MT	3.65 (.26)	3.52 (.24)	.60
MT	3.45 (.30)	3.05 (.27)	.14
AMB	3.22 (.31)	3.22 (.28)	.99
*Change in health*			
Whole sample	3.68 (.09)	3.56 (.08)	.17
AMB + MT	3.67 (.23)	3.48 (.22)	.40
MT	3.85 (.26)	3.43 (.25)	.10
AMB	3.58 (.27)	3.53 (.26)	.86
*Overall health*			
Whole sample	3.79 (.08)	3.70 (.09)	.30
AMB + MT	3.72 (.21)	3.81 (.24)	.72
MT	3.97 (.24)	3.55 (.27)	.13
AMB	3.41 (.25)	3.36 (.28)	.87
*Social support*			
Whole sample	4.00 (.10)	4.01 (.11)	.94
AMB + MT	4.23 (.27)	4.15 (.30)	.79
MT	4.10 (.31)	3.92 (.34)	.59
AMB	4.10 (.32)	4.00 (.35)	.78
*Quality of life*			
Whole sample	4.04 (.06)	3.99 (.07)	.50
AMB + MT	4.33 (.17)	4.27 (.17)	.73
MT	4.39 (.19)	4.27 (.20)	.55
AMB	3.94 (.20)	4.13 (.20)	.35
*Anxiety*			
Whole sample	1.87 (.06)	1.83 (.05)	.41
AMB + MT	1.75 (.15)	1.70 (.14)	.74
MT	1.90 (.17)	1.83 (.16)	.66
AMB	1.86 (.17)	1.72 (.17)	.39
*Depression*			
Whole sample	1.69 (.05)	1.71 (.05)	.73
AMB + MT	1.67 (.14)	1.69 (.13)	.86
MT	1.92 (.15)	1.75 (.15)	.26
AMB	1.90 (.16)	1.71 (.16)	.22
*Loneliness*			
Whole sample	2.30 (.06)	2.32 (.05)	.64
AMB + MT	2.51 (.17)	2.43 (.14)	.59
MT	2.38 (.20)	2.22 (.16)	.34
AMB	2.22 (.20)	2.29 (.16)	.66
*Subjective vitality*			
Whole sample	4.62 (.11)	4.47 (.12)	.21
AMB + MT	4.20 (.29)	4.43 (.32)	.46
MT	4.63 (.33)	4.46 (.36)	.63
AMB	3.77 (.34)	4.46 (.37)	.07

Note: AMB + MT = Ambassadors plus motivation training; MT = Motivation training without ambassadors AMB = Ambassadors without motivation training. The no ambassadors nor motivation training (AMB or MT) condition was the reference group.

We also examined changes in all mental health and well-being variables across all three time points in the main experimental (AMB + MT) condition only. There were no changes over time in the outcomes, with one exception. Participants in this group reported reductions in perceived fitness from baseline (*M *= 3.53; *SE *= .15) to post-intervention (*M *= 3.13; *SE *= .15) and 6-month follow-up (*M *= 2.92; *SE *= .15).

It was apparent from the interviews with the walkers that the group-based format could function as both a barrier and enabler for increasing and maintaining physical activity. Several walkers noted that group walks did not work well for older adults. For example, one participant stated:

… varying degree of oldness have different pace of walking … other people like to walk quickly. That wouldn’t suit all different speeds. It’s never going to work with older people. (Lydia, age 71, drop-out, MT)

Another walker who dropped out stated:

I just felt maybe the group was the wrong target. (Harriet, age 68, drop-out, no AMB or MT)

Reasons for this argument thus seemed to be associated with different capabilities of walkers within a group, but also due to competing demands, busy schedules and challenges identifying times to walk that were suitable for all. Accordingly, several participants mentioned that they preferred to walk alone:

I would like to go for my walk and I can, but that doesn’t always suit other people … that’s why I think sometimes I’m better just to take off on my own. I don’t know that I’m so great in group things. (Sally, age 80, adherer, AMB + MT)

A key focus of the motivation training delivered to walkers was to promote self-determined reasons to walk to facilitate sustained regular walking. Some walkers reported reasons to participate in group walks that aligned with such motivation (see Table 1). Aligned with SDT, promoting competence need satisfaction was one of the key mechanisms via which we aimed to promote more self-determined motivation. Several participants mentioned how group walks increased their confidence:

… I’m sure the safety aspect would come in to it more because walking in a group, there’s always someone there if you do trip. (Selma, age 73, adherer, no AMB or MT)

The pedometer served as an important motivator that helped participants to focus on autonomous reasons to be active (e.g. personal interest and accomplishment):

I still use it. As a matter of fact, I’ve got it on right now and it was a matter of great interest as to how many steps I’d done in one day and I try to increase them if I could. (Elizabeth, age 87, adherer, AMB + MT)

An adhering participant from a different village (AMB + MT) suggested that having the pedometer was useful in terms of raising awareness of when to be active and when to rest:

Later on, in the day, I’ll have a look and see how many steps I’ve done, and I think ‘Well, that’s great. I deserve to be able to sit down now. I’ve done a fair amount of walking’. So, I guess it makes you aware of both your resting time and your walking or activity time. (Selma, age 73, adherer, no AMB or MT)

Ambassadors and managers also agreed that the pedometers served an important motivational function.

More broadly, the programme was successful in helping to raise awareness of the importance of being active and reducing sedentary behaviour:

I'm finally understanding what they talk about when they talk about people becoming couch potatoes, not because I'm becoming one, but I've become aware that when you sit, you actually feel tired to the point, if I'm watching – I love British mysteries, you will laugh, they usually go for about two hours but every half hour, I get up and do some exercises. (Susie, age 73, adherer, no AMB or MT)

A key focus of the ambassadors’ role was to promote the adoption of behaviour change techniques (BCTs) by the walkers to promote sustained behaviour change. BCTs are observable and replicable active ingredients of interventions (Michie & Johnston, 2012), such as implementation intentions, self-monitoring and goal setting. The quotes presented in Table 1 provided some evidence that the ambassadors were using the ten motivational themes as a means to apply the need-supportive strategies during the intervention, and, as such, the table categorises quotes by theme, as well as by the relevant RE-AIM dimensions. Most predominantly, the interviews provided considerable evidence that the walkers were made to feel like valued members of the group and this feeling was evidenced by the walkers reflecting on how they would look out for one another. There were also a number of quotes that highlighted what the walkers found most useful in terms of maintaining walking, indicating that the walkers had developed new habits and behavioural self-awareness that helped support on-going walking. The role of experiencing accomplishment and a sense of competence was also expressed as an important, motivating factor.

### Adoption

We directly contacted 10 retirement village providers in WA. Of those, seven providers (most of which consisted of many villages) consented to take part in the intervention. On average, 23% of the villages from each provider agreed to take part. Of the 14 villages that consented to take part, one dropped out because it proved impracticable to schedule a training date for participants, as many of the consenting residents were travelling for extended periods of time. In terms of ambassadors, a total of 36 participants expressed interest in taking on this role. Of these, 11 dropped out (*n *= 5 for medical reasons, and *n *= 6 due to perceived lack of time). Further, 17 who had expressed interest in becoming ambassadors became walkers for a range of reasons (including lack of time, medical reasons, and insufficient number of willing ambassadors in the village). This left a total of eight participants (*n *= 7 female) who completed the ambassador role, representing 22% of those who showed initial interest. Ambassadors were residents in the villages aged 70–83 years old (*M *= 72.29; *SD *= 5.06). Three were on resident committees in their respective villages. All of them had previous volunteering experience (*M* years = 14.25; *SD *= 20.74) and six had leadership experience (*M* years = 21.50; *SD* = 21.35).

The ambassadors who were trained reported high levels of satisfaction with the training they received (*M *= 5.74; *SD *= 1.03, out of a total possible score of 7). Further, on average the ambassadors liked being ambassadors (*M *= 3.50 out of 5; *SD *= 1.51), had reasonable confidence (*M *= 3.29 out of 5; *SD *= 1.25) and reported moderate perceptions of effectiveness (*M *= 3.00 out of 5; *SD *= 1.00).

The analysis of the interviews revealed that the ambassadors expressed high levels of self-determined motivation to engage in the role:

I was excited. I thought it was a great and incredible idea to get people involved to do that. (Bernadette, age 81, ambassador, drop-out, AMB + MT)

I enjoyed walking and I liked the idea to try to get more people here walking. (Anna, age 74, ambassador, completer, AMB + MT)

In the same vein, one of the other ambassadors commented on the satisfaction of helping others:

… the walk was good for me because I enjoy walking and I think the sense of being able to help people made me feel good. It made me feel good that I could do that. (Bev, age 70, ambassador, completer, AMB + MT)

However, it was also clear from interviews with managers and two of the ambassadors that the ambassador role was perceived as a big commitment that many were not willing to take on. One of the village managers noted that it was a good idea to appoint ambassadors for the programme, but that those who could potentially act in that role struggled to commit to the role:

I think having a resident leading is great ‘cause it means someone like myself doesn’t have to be always pushing that and they take a bit of self-ownership, but I just think again, great idea and something that I would love to have … .but there’s always someone away and no one wants to commit to being that person at the same time every week. (Emma, manager, AMB + MT)

The interviewed ambassador who dropped out from the programme (due to injury) noted that being an ambassador was an (unexpectedly) big commitment. However, interestingly, in one of the villages where no one wanted to formally take on the role of ambassador, informal peer leadership emerged:

No one was willing to step up and take on that – with that title, but [name] seemed to be the one that was doing that job … she was the one that sort of was saying ‘come on, we’re going for a walk tomorrow and you’re coming’ and things like that. (Charlotte, manager, MT)

The AMB + MT ambassador interviewed who did take on the role and completed the programme demonstrated intrinsic motivation for the role, but expressed disappointment that the group dismantled after a while:

I found it very enjoyable and I was very disappointed that we couldn’t get people motivated enough to keep going. (Emma, age 74, ambassador, completer, AMB + MT)

In contrast, the third ambassador who was interviewed described the experience of being an ambassador as relatively easy:

As far as my group goes, they made it easy for me … it wasn’t a hard group to mentor. The ambassador as a role, it wasn’t a hard role. (Bev, AMB + MT)

From the walkers’ perspective, ambassador engagement was variable. Some noted limited or no contact with their ambassadors, that the ambassador was not around for long, or that the ambassador did not exhibit sufficient flexibility in scheduling walks. One of the walkers noted:

Our ambassador likes going earlier and then in the winter time, sort of, I didn’t want to get up that early so that’s why it just fell apart and I said ‘Look this isn’t working, I’m going to walk on my own time. (Ida, age 74, AMB + MT)

One of the managers implied that it might be important to involve the administration (managers) in the selection of ambassadors to identify those most suitable, as she noted that the managers would know of:

… any challenges they [the ambassadors] might face that might impede the process. (Tracy, manager, AMB + MT)

### Implementation

A total of 57% of walkers who consented to take part completed the face-to-face training; the remaining participants who could not attend face-to-face training received a training DVD and the training folder distributed to the participants who attended the face-to-face training. The walker training was well received with a mean rating on the training acceptability scale of 5.97 (*SD *= .81) out of a maximum score of 7. Overall intervention satisfaction in the AMB + MT condition was also rated favourably by the participants (*M* = 5.79; *SD* = 1.01). Sixty walkers (52%) provided logbook data. Of those who returned the log-books, a mean of 14.79 (*SD *= 1.98) weeks of the 16-week programme were completed. Action planning sheets were completed by 14 walkers (12%). Of the participants who completed action planning sheets, 72% of sheets were completed.

The interviews with the ambassadors and walkers revealed some evidence that the ambassadors implemented some of the motivational strategies they were taught (Table 1). One ambassador noted that these motivational strategies were somewhat useful, but were not implemented continually:

I took a little bit on board, I wouldn’t say I used them all the time but when I started, I did. (Anna, age 74, ambassador, completer, AMB + MT)

Interestingly, some of the walkers did not recognise that the strategies the ambassadors were implementing were relevant to motivation. However, their testaments revealed that the ambassadors did use motivational strategies taught in their training. For example, those designed to increase feelings of relatedness (e.g. getting to know the walkers and helping them feel at ease) were deliberately intended motivational strategies, even if not perceived as such. There was also considerable evidence that the walkers were using the behaviour change techniques that they were taught and recognised these as useful to keep up their walking. Use of the self-monitoring tools was most prominent in this regard (see Table 1).

### Maintenance

Among the managers who were interviewed, there were varied reports of the extent to which they perceived that participants in the trial continued to walk following the end of the programme. The completing ambassador expressed how enjoyment associated with walking in a group kept that group walking together following the end of the programme (see Table 1). The interviews revealed that many walkers had identified what would help them continue their walking and set new goals. Several participants noted that routine was important for engaging in daily walks, and they had identified specific walking routes that would help them to continue. These included ongoing use of self-monitoring and capitalising on the social support of others to facilitate maintenance.

For those who did not walk at the time of the interviews, reasons included family commitments and grieving. The participant who was grieving had struggled to adhere throughout the programme but remained involved. In terms of her maintenance she noted:

Well I have to admit that I am not [walking]. I’ve had a very difficult time and my husband’s been very ill and he passed away a couple of weeks ago … Well, it made it all rather too difficult for me and I haven’t done it lately, but I will try and get back to it. (Isobel, age 84, challenged but remained, AMB + MT)

The manager of a different village noticed people taking part in the programme 6 months later:

I see about seven people that participated that were at the end of the trial, I see those seven people out walking pretty regularly including two people that were in a group together that I haven’t seen walking together previously. (Tracy, manager, AMB + MT)

The same manager noted how the trial has encouraged people who did not take part in the trial to start walking, implying a possible social contagion effect (Christakis & Fowler, 2013):

I see probably another 12–13 non-trial participants that walk now that weren’t walking 12 months ago, but they were non-participating people. What you probably found was that people were inspired in the RiAT trial, so other people started thinking ‘Oh, I better get off my butt and go and do something’, and they may have been people that didn’t commit for a whole lot of reasons, but I think that’s pretty interesting. (Tracy, manager, AMB + MT)

Another manager explained how the walkers had been very eager to walk at the beginning, but that deteriorating health negatively impacted participation in the longer term:

Unfortunately, we got residents who were sick, and their health deteriorated. So, that sort of, that impacted on their ability to go out and go walking so it’s hard to say if they hadn’t have had the illnesses whether they would have kept going or not. (Charlotte, manager, MT)

## Discussion

The aim of the present study was to examine the feasibility, acceptability and viability of a group-based peer-led motivationally-embellished walking intervention, designed to increase walking, reduce sitting and improve mental health in older adults in retirement villages.

### Trial feasibility

Significant issues relating to trial feasibility are likely to have compromised the efficacy of this trial. Specifically, we were unable to recruit sufficient ambassadors (peer leaders), which resulted in a cluster quasi-experimental research design, instead of the intended cluster randomised controlled design. The changed design led to the creation of two additional conditions which, coupled with the general struggle to recruit sufficient number of walkers, resulted in an under-powered trial. The qualitative evidence we gathered revealed that a major reason for lack of engagement with the trial was the perceived burden of the number and frequency of assessments the participants were asked to complete to evaluate the trial. Lingler, Schmidt, Gentry, Hu, and Terhorst (2014) showed in a sample of 134 older adults who were research volunteers that perceived research burden was negatively related to the likelihood of enrolling in a research study. Another finding emerging from the interviews suggested that participants wanted more contact with the research team in the lead up to the start of the intervention. These findings point to the need for careful planning of contact with participants pre-intervention, particularly if multiple sites are included, and managing participant expectations regarding the availability of the research team. In a systematic review of recruiting participants to walking studies, Foster et al. (2011) discussed the possible need for training of recruiters and engaging future participants in the recruitment process.

### Reach

The Residents in Action Trial reached a relatively small proportion of eligible residents in the participating retirement villages suggesting that more effective recruitment strategies need to be identified for future research. The interview findings showed that a large proportion of eligible residents (i.e. those who needed to increase their walking) were those who were generally not engaged in other social or physical activities in the village. This suggests that the addition of one-to-one targeted recruitment approaches may be needed to recruit more eligible and representative study participants, where resources allow (Cooke & Jones, 2017). Foster et al. (2011) identified in their systematic review that studies have utilised passive, active, and combined methods of recruitment to walking studies. Trials using only active methods (e.g. researchers contact potential participants directly, such as through phone calls) were less frequent. From 47 studies reviewed, 31 used passive methods, including mail drops and posters. In the current study, we used a mix of active and passive recruitment methods. Foster et al. also concluded that approaches for recruiting participants into walking studies are likely to require time and resources. In the case of our study, greater support from some of the village managers would have facilitated opportunities for more direct recruitment. A challenge we faced was that the level of support for recruitment from managers varied considerably across villages.

It is also noteworthy that at both ambassador and walker levels, the majority of participants were female (approximately 80%). While this is consistent with trends seen in other interventions that aim to engage older adults in health and PA interventions (e.g. Gavarkovs, Burke, & Petrella, 2016) it is an issue worthy of attention in future research studies. Innovative approaches such as male-focused programmes that consider masculine ideals and gender influences to engage men are possible ways forward to better reach and engage men in health promotive PA (Bottorff et al., 2015).

### Efficacy

The retention rate (92%) in the intervention was excellent in comparison to many other similar physical activity trials (Ball, Abbott, Wilson, Chisholm, & Sahlqvist, 2017; Jancey et al., 2007). Further, no adverse events were reported suggesting that this trial is safe. Moreover, participants in the main experimental condition (i.e. AMB + MT) increased their step counts step counts by 14% from baseline to post-intervention. This rate exceeds Fitzsimons et al. (2013) and is comparable to Rosenberg et al. (2015) to step count increases reported in uncontrolled feasibility studies with older adults, and randomised controlled trials with older adults (Croteau, Richeson, Farmer, & Jones, 2007; Harris et al., 2015). Unfortunately, due to the trial feasibility issues we experienced, we did not manage to secure sufficient activPAL data post-intervention to examine changes over time in the other three conditions. In regard to the mental health and well-being outcomes, when comparing the groups from baseline to post-interventions, we identified a reduction in the physical health composite score for the MT participants from baseline to post-intervention. However, it is important to note that due to the aforementioned issues with trial feasibility, the study was underpowered to detect any differences between the groups.

There was one other unexpected finding when examining changes in perceived fitness in the AMB + MT group, with scores for this variable decreasing over time. This might be because, as participants tried to increase their physical activity, they became more accurate in their estimation of fitness levels. This trend could also be due to age-related decline and confounding health issues.

### Adoption

Far fewer ambassadors than expected were recruited for the study. The perceived commitment that the role entailed appeared to be an important barrier to participation. Limited time to act as a peer leader has been identified as an important barrier in other (qualitative) studies (Washburn, Cornell, Traywick, Felix, & Phillips, 2017). While ambassadors reported positively about their training and intrinsic motivation for the role, and reasonably favourably on their confidence and perceptions of effectiveness, the ambassadorial role was seen as demanding. Moreover, some walkers felt that ambassadors needed to operate with greater flexibility, possibly adding more demands to the role. It is clear that future studies using walk ambassadors in retirement villages will need to consider very carefully how this role is supported, what the demands are, and how best to operate what, in theory, should be a highly positive resource. These factors could be gleaned from further ‘patient and public involvement’ work prior to the study starting.

### Implementation

Over half of the walkers attended face-to-face training, and this was well received. Moreover, half of the walkers provided data from their logbook and, where this was provided, most weeks were completed. However, action planning sheets were much less used. This might relate to the reported ‘paperwork’ burden. It is challenging to achieve the balance between manageable participant burden and participant support. Action planning is seen as a potentially highly beneficial method for supporting behaviour change and was utilised in this trial (Hagger et al., 2016). We were not successful in promoting this behaviour change technique amongst the participants. Further input from participants might reveal additional strategies to make completion of such planning activities more personally-relevant and appealing.

The motivational strategies appeared to be implemented, at least in part, by the ambassadors. Some walkers did not recognise some strategies explicitly as motivation-supportive. This makes sense, given that the lay understanding of what it means to be motivationally supportive is likely to be quite different from what we, as researchers, know to result in higher quality motivation. Therefore, it is not really an issue as to whether or not the strategies were recognised as being motivationally supportive, it is more important to identify that they had this effect. This finding highlights the importance of including interview questions in process evaluations in SDT-based studies revolving around the mechanisms which are directly impacted by a motivationally supportive environment (i.e. feelings of need satisfaction) rather than motivation per se. Even if some walkers did not think some strategies were motivation relevant (e.g. social connectedness through walking conversations), our findings show that such strategies reinforced motivation for walking.

### Maintenance

Results suggested that facilitators to walking maintenance included the use of self-monitoring, goal setting, social support, and having a routine, including utilising familiar walking routes. Self-monitoring and social support are well-recognised behaviour change techniques (Michie et al., 2013). How long walkers will utilise pedometers or some other form of self-monitoring is unknown, but the evidence does suggest that such self-monitoring is effective, at least in the short to medium term.

The use of social support appears to be key for this population. Using social strategies and ensuring that the need for social relatedness is met, may be key to the maintenance of walking and other physical activities. Not only will social connectedness help with issues such as scheduling and daily adoption of walking (i.e. getting started), it may also assist with enhanced perceptions of safety. Moreover, observation of others walking may be helpful as it was noted that a social contagion effect may have been in operation in some villages, in that residents not participating in the trial were inspired by watching the study participants and started walking in their own groups. Barriers to participation included family commitments, poor health, and grieving. The latter may also link to social support as individuals coping with the grieving process could be supported into a group walking programme as a means of coping with bereavement.

### Study limitations and future research directions

The change in the research design and the low statistical power are two of the main limitations of the study. Another limitation was the relatively high proportion of individuals who were not physically inactive (as established by accelerometer data), despite self-reporting that they were. The comprehensive assessment of the various aspects of the trial in this paper offers researchers in this area a magnitude of information regarding what worked and what can be done to avoid some of the problems we experienced in the future (e.g. careful selection of number of participant assessments perhaps by having a priori consultation phase, review level of commitment of ambassadors prior to the start of the training, establish tangible support from village managers before enrolling a village to a trial). The qualitative evidence suggests that peer support is critical, but that groups may not be the most appropriate format to achieve this for some participants. It is recommended that other approaches are also investigated. For example, a recent systematic review and meta-analysis provided evidence for the effectiveness of dyadic approaches to physical activity promotion (Carr et al., 2018). Walking in pairs as opposed to walking in groups might overcome some of the logistical problems described by participants, such as finding convenient times for group walks or identifying groups of walkers with similar walking ability.

### Conclusions

Among those walkers and ambassadors who did take part in the study, results suggested that they enjoyed the programme and found it useful in terms of becoming more active and making social connections. Hence, the premise of delivering a walking intervention via motivationally-trained peer leaders (ambassadors) and walkers should be further tested in future trials that address the organisational and research assessment challenges we experienced.

## Supplementary Material

Supplemental Material

## ORCID

*Cecilie Thøgersen-Ntoumani* http://orcid.org/0000-0003-0255-1263

*Eleanor Quested* http://orcid.org/0000-0001-8955-8809

*Stuart J.H. Biddle* http://orcid.org/0000-0002-7663-6895

*Marlene Kritz* http://orcid.org/0000-0002-4706-4891

*Jenny Olson* http://orcid.org/0000-0003-2777-3794

*Elissa Burton* http://orcid.org/0000-0001-6470-8305

*Ester Cerin* http://orcid.org/0000-0002-7599-165X

*Keith D. Hill* http://orcid.org/0000-0002-2191-0308

*Joanne McVeigh* http://orcid.org/0000-0002-2446-3814

*Nikos Ntoumanis* http://orcid.org/0000-0001-7122-3795
