# Supplementary material for: Trial feasibility and process evaluation of a motivationally-embellished group peer led walking intervention in retirement villages using the RE-AIM framework: the residents in action trial (RiAT)
Source: Health Psychol Behav Med. 2019 Jun 17;7(1):202–33. doi: 10.1080/21642850.2019.1629934 (PMC8114369; doi:10.1080/21642850.2019.1629934)
Supplement: Supplemental Material [file RHPB_A_1629934_SM0953.docx]

**Walker interview guide (individual, pair based and focus groups)**

- Why did you decide to take part in the RiAT program?
- Can you describe your experience of taking part at the beginning of the program?
- Can you describe your experience of taking part in a research trial?
- What did you think about
  - The walk maps?
  - The training workshops?
  - The material included in the training folders?
  - The walk groups?
  - The pedometer?
- What made it challenging for you to take part in the program?
  - How did you deal with those challenges?
- How do you feel about the program now that you have completed it?
- What are your plans in terms of walking in the future?
- How do you think the program could be improved?

**Ambassador interview guide**

- Why did you decide to take part in the RiAT program?
- What did you think about
  - The walk maps?
  - The training workshops?
  - The material included in the training folders?
  - The walk groups?
  - The pedometer?
  - What, if anything, would you change? Why?
- Which, if any, elements of the program did you find it difficult to engage with?
- Can you describe your experience of taking part in a research trial?
- What was your experience of trying to use the motivation strategies (ie., the training) with the walkers?
- If we were to conduct the study again, what do you think we should do to enable us to recruit more physically inactive residents?
- How can we improve the program for the future?

**Village manager interview guide**

- Can you please tell me more about your role at the village?
- What was the village’s previous experience with physical activity programmes before you agreed/disagreed to offer the RiAT programme to residents?
- What were your first thoughts about residents taking part in RiAT?
- What were the reasons why you agreed/disagreed for the village to get involved in the RiAT programme?

What factors do you think contributed to some of the residents signing up for the program?

- What do you perceive were the possible reasons for the lack of engagement in the program by some residents in your village?
- Did you feel you were in a position to provide support to the program? If so, how?

If this study was conducted again, based on your experience as village manager, what suggestions would you make to improve

How the research team recruit/engage retirement villages

How the research team could recruit/engage ‘*inactive*’ residents

What was your impression in terms of how much the participants in the program engaged with the different aspects of the programme?

What impact do you think the RiAT programme have on the residents who took part in the program? Can you provide me with some examples?

What impact, if any, did the RiAT programme have on residents *who did not* participate in programme?

Do you think that the format of the program could be improved?

What are your thoughts about RiAT now the program is complete?

- Since completion of the RiAT program, have you seen any residents who participated in the program walking regularly on their own or as part of a group?
- Lastly, what do you think are the critical factors to help make RiAT or similar walking programs sustainable in the long term within your village?
